# HDAC Inhibition in Vascular Endothelial Cells Regulates the Expression of ncRNAs

**DOI:** 10.3390/ncrna2020004

**Published:** 2016-05-25

**Authors:** Haloom Rafehi, Assam El-Osta

**Affiliations:** 1 Epigenetics in Human Health and Disease Laboratory, Baker IDI Heart and Diabetes Institute, 75 Commercial Road, The Alfred Medical Research and Education Precinct, Melbourne, VIC 3004, Australia; haloom.rafehi@bakeridi.edu.au; 2 Department of Pathology, The University of Melbourne, Parkville, VIC 3052, Australia; 3 Faculty of Medicine, Monash University, Melbourne, VIC 3168, Australia

**Keywords:** non-coding RNA, epigenetics, HDAC inhibitors, histone acetylation, histone methylation, endothelial cells, vascular

## Abstract

While clinical and pre-clinical trials indicate efficacy of histone deacetylase (HDAC) inhibitors in disease mediated by dynamic lysine modification, the impact on the expression of non-coding RNAs (ncRNAs) remains poorly understood. In this study, we investigate high throughput RNA sequencing data derived from primary human endothelial cells stimulated with HDAC inhibitors suberanilohydroxamic acid (SAHA) and Trichostatin A (TSA). We observe robust regulation of ncRNA expression. Integration of gene expression data with histone 3 lysine 9 and 14 acetylation (H3K9/14ac) and histone 3 lysine 4 trimethylation (H3K4me3) datasets identified complex and class-specific expression of ncRNAs. We show that EP300 target genes are subject to histone deacetylation at their promoter following HDAC inhibition. This deacetylation drives suppression of protein-coding genes. However, long intergenic non-coding RNAs (lincRNAs) regulated by EP300 are activated following HDAC inhibition, despite histone deacetylation. This increased expression was driven by increased H3K4me3 at the gene promoter. For example, elevated promoter H3K4me3 increased lincRNA *MALAT1* expression despite broad EP300-associated histone deacetylation. In conclusion, we show that HDAC inhibitors regulate the expression of ncRNA by complex and class-specific epigenetic mechanisms.

## 1. Introduction

Once thought to be functionally inert, non-coding RNAs (ncRNAs) are now considered key regulators of chromatin structure and gene function [1]. They represent the majority of the human transcriptome by number [2]. They are essential for physiological development and more recently have been implicated in human disease, including cancer and heart disease [3,4].

Importantly, ncRNAs are highly diverse in their structure and function and include both short and long ncRNAs. Long ncRNAs are divided into multiple subclasses based on their genomic locations and DNA sequence [5]. For example, long intergenic non-coding RNAs (lincRNA) are transcribed from segments of DNA located outside of protein-coding genes. Other classes of long ncRNA include sense intronic genes that are transcribed from introns of protein-coding genes without overlapping exons. In contrast, sense overlapping ncRNA transcripts contain a coding gene with its intron on the same strand, whereas antisense ncRNA are transcribed from the opposite strand of a protein-coding gene and can span across both exons and introns. Pseudogenes are also classified as long ncRNAs, non-coding duplications of functional protein-coding genes with mutations that result in disruptions to the open reading frame (ORF). Some, though not all, pseudogenes have promoters and can transcribe ncRNA. Long ncRNAs that cannot be classified and do not contain an ORF are referred to as processed transcripts [2].

Recently, a study of 111 reference epigenomes by the National Institute of Health (NIH) Roadmap Epigenomics Project identified complex interactions of histone modifications with lincRNA expression [6]. Chromatin modifications at lincRNAs were highly tissue-specific: histone 3 lysine 4 monomethylation (H3K4me1) was more variable across tissue types compared to histone 3 lysine 4 trimethylation (H3K4me3). Histone 3 lysine 27 trimethylation (H3K27me3) is a key regulator of lincRNA gene silencing during lineage commitment. Histone acetylation, which is written by histone acetyltransferases (HATs) and erased by histone deacetylases (HDACs), is enriched at active transcription start sites. This includes both histone 3 lysine 9 acetylation (H3K9ac) and histone 3 lysine 27 acetylation H3K27ac. These profiles suggest that the expression of ncRNAs can potentially be targeted by pharmacological manipulation of the epigenome.

Histone deacetylase (HDAC) inhibitors are a diverse group of clinically used pharmacological compounds [7,8]. While the paradigmatic mechanism of action suggests increased histone acetylation mediating gene activation, more recent profiling studies show broad histone deacetylation at gene promoters implicated in the regulation of gene suppression [9]. HDAC inhibitors are also known to regulate other histone modifications, including trimethylation of H3K4me3, which is a key driver of gene expression [9,10]. However, the extent to which HDAC inhibitors can control the expression of ncRNA classes remains unclear.

To investigate gene regulation of ncRNA by HDAC inhibition, we examined gene expression changes mediated by histone acetylation (H3K9/14ac) and histone methylation (H3K4me3) in primary human aortic endothelial cells (HAECs) stimulated with trichostatin A (TSA) and the structurally related and clinically used suberanilohydroxamic acid (SAHA, also known as vorinostat) [9]. Recent evidence indicates that ncRNAs are important regulators of endothelial cell function [11]. For example, the lincRNA metastasis associated lung adenocarcinoma transcript 1 (*MALAT1)* regulates blood vessel growth and *MALAT1* inhibition prevents human endothelial cell proliferation and reduces vascular growth [11]. Our findings confirm that HDAC inhibition regulates the expression of non-coding RNAs dependent on chromatin modifications.

## 2. Results

### 2.1. Pharmacological Inhibitors of HDAC Activity Regulate the Expression of ncRNAs

**Figure 1:**
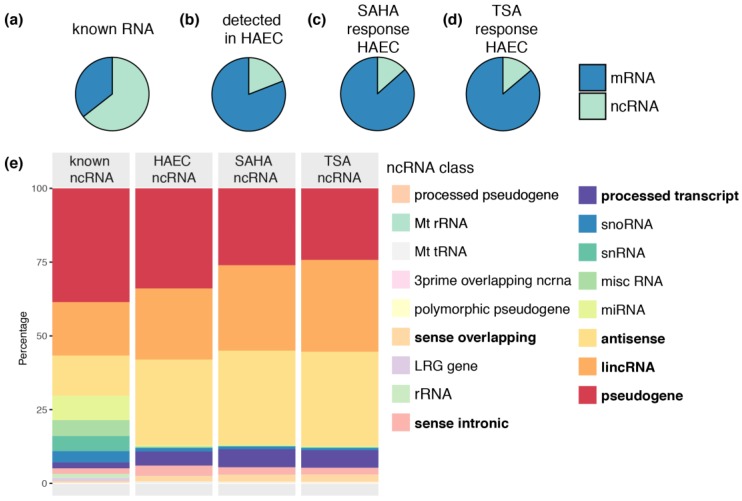
HDAC inhibitors regulate the expression of ncRNAs. Pie charts comparing (**a**) relative number of known mRNA and ncRNA; (**b**) relative number of mRNA and ncRNA genes detected in HAECs by RNA-seq, and relative number of mRNA and ncRNA differentially expressed in HAECs stimulated with HDAC inhibitors (**c**) SAHA and (**d**) TSA (FDR *p* value < 0.05); (**e**) A stacked bar chart compares percentages of known ncRNA classes, ncRNA classes detected in HAECs by RNA-seq, and the percentage of ncRNA classes differentially expressed by SAHA and TSA in HAECs (FDR *p* value < 0.05). Abbreviations: Mt rRNA: mitochondrial ribosomal RNA; Mt tRNA: mitochondrial transfer RNA; LRG gene: Locus Reference Genomic gene; rRNA: ribosomal RNA; snoRNA: Small nucleolar RNA; snRNA: small nuclear RNA; misc RNA: Miscellaneous RNA; miRNA: microRNA; lincRNA: long intergenic non-coding RNA.

To investigate whether HDAC inhibitors can broadly regulate the expression of ncRNA genes, we re-analysed publically available RNA-seq and H3K9/14ac and H3K4me3 chromatin immunoprecipitation sequencing (ChIP-seq) data from primary HAECs stimulated with the hydroxamic acid HDAC inhibitors SAHA and TSA. While ncRNAs represent approximately 64% of all known and predicted genes (Figure 1a), over 20% of the transcripts detected in HAECs are ncRNA (Figure 1b). Stimulation of HAECs with SAHA (Figure 1c) and TSA (Figure 1d) induced differential expression of ncRNAs.

Pseudogenes, lincRNA, antisense RNA, processed transcripts, sense overlapping and sense intronic transcripts were highly represented in the dataset (Figure 1e). These six classes were included for further analysis. Other transcript classes, such as miRNA, were not adequately captured by this protocol and were excluded from further class-specific analysis of RNA-seq data.

We identify robust changes in gene expression induced by SAHA and TSA in all gene classes (Figure S1a). In response to HDAC inhibition, 1498 ncRNA genes were differentially expressed by SAHA and 1274 ncRNA genes by TSA (false discovery rate (FDR) *p* value < 0.05) (Figure S1b). Consistent with previous reports [9], gene expression changes induced by SAHA are strongly correlated with gene expression changes in response to TSA (Figure S1c).

### 2.2. Chromatin Modifications Drive ncRNA Gene Activation and Suppression

**Figure 2:**
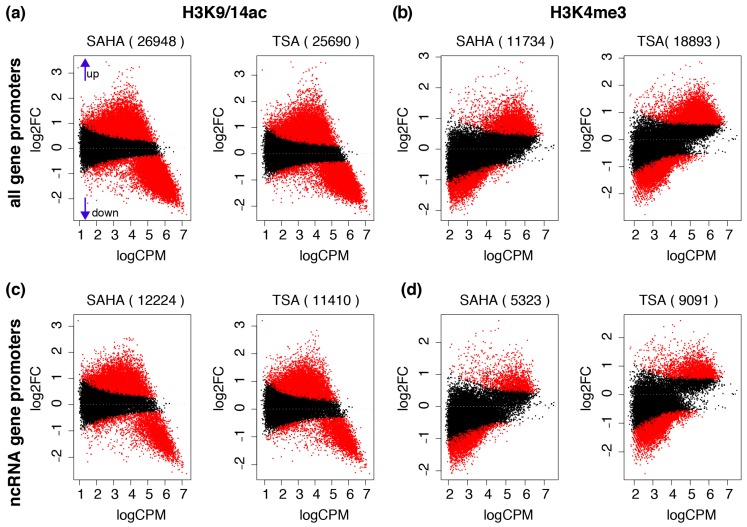
Dynamic histone acetylation changes at the ncRNA promoters by HDAC inhibition. Mean-Average (MA) plots show the fold changes (logFC, log2 of the fold change) and the relative read concentration (logCPM) for histone acetylation (H3K9/14ac) at (**a**) all genes and (**b**) ncRNA gene promoters, as well as histone methylation at (**c**) all genes and (**d**) ncRNA gene promoters. Red points indicates FDR *p* value < 0.05, and black indicates FDR *p* value > 0.05. The number of genes FDR < 0.05 is shown in parenthesis on top of each plot.

Because HDAC inhibitors alter histone modifications at gene promoters of coding genes [9], we investigated whether SAHA and TSA induce changes to H3K9/14ac and H3K4me3 at ncRNA gene promoters. We observe strong histone acetylation and deacetylation at gene promoters (Figure 2a), including ncRNA genes (Figure 2b). In particular, strong histone deacetylation was observed at promoters of genes that were highly acetylated prior to stimulation with SAHA or TSA. Promoters with a low to moderate level of histone acetylation prior to stimulation are subject to increased histone acetylation. Furthermore, we identify strong changes to H3K4me3 by SAHA at gene promoters (Figure 2c), including ncRNA gene promoters (Figure 2d). In contrast to histone acetylation, we found increased H3K4me3 at gene promoters that have a moderate to high level of H3K4me3 prior to HDAC inhibition, whereas decreased H3K4me3 is more likely to occur at genes with low promoter H3K4me3 in untreated cells.

**Figure 3:**
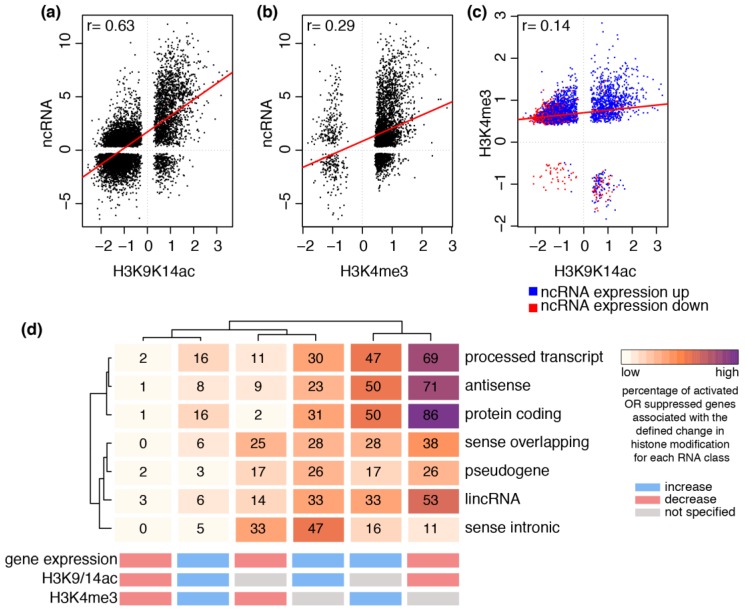
Epigenetic regulation by SAHA is dependent on ncRNA type. Scatterplots plots of the fold changes (logFC) show the correlation between ncRNA expression (FDR *p* value < 0.05) and corresponding promoter (**a**) H3K9/14ac and (**b**) H3K4me3 (FDR *p* value < 0.05) in response to SAHA stimulation; (**c**) A scatterplot of the fold changes (logFC) shows the correlation between differential promoter H3K9/14ac and H3K4me3 (FDR *p* value < 0.05) for ncRNA genes that are either activated (blue) or suppressed (red) (FDR *p* value < 0.05). Linear model is shown in red and the Pearson’s correlation value is reported for each plot; (**d**) A heatmap showing the percentage of activated or suppressed genes that overlap with increased or decreased histone modification (defined on horizontal axis) for protein-coding genes and six ncRNA classes (vertical axis).

Because the promoters of ncRNA genes are subject to altered chromatin modifications by HDAC inhibitors, we investigated whether H3K9/14ac and H3K4me3 were associated with differential gene expression. To do this, we integrated the expression of ncRNA transcripts with H3K9/14ac and H3K4me3 profiles at gene promoters in response to SAHA and TSA stimulation in HAECs. We identify a positive correlation for gene expression and histone acetylation in response to SAHA stimulation (Pearson’s *r* = 0.63) (Figure 3a). A consistent trend was observed for HAECs stimulated with TSA (Pearson’s *r* = 0.68) (Figure S2a). Differential H3K4me3 integrated with ncRNA gene expression changes show a weak correlation in response to SAHA (Pearson’s *r* = 0.29) (Figure 3b) and TSA (Pearson’s *r* = 0.24) (Figure S2b). However, increased histone methylation was associated with 2814 activated ncRNA genes. Furthermore, while 923 genes were associated with both increased H3K9/14ac and H3K4me3, we identify 2499 genes with increased H3K4me3 and reduced H3K9/14ac (Figure 3c). A similar trend was observed in response to TSA stimulation (Figure S2c).

To investigate the relationship between histone modifications and gene expression in more detail, we determined the percentage of activated or suppressed genes associated with increased or decreased histone modifications for the different ncRNA groups (Figure 3d). We selected six ncRNA classes that were detected by RNA-seq: pseudogenes, lincRNA, antisense RNA, processed transcripts, sense overlapping and sense intronic transcripts. Protein-coding genes were analysed as a reference group. The results are represented as the percentage of activated or suppressed genes that are associated with the specified histone modification change. Corresponding odds ratios based on Fisher’s test are summarised in Figure S3.

We show that histone modifications of processed transcripts and antisense genes correlate strongly with protein-coding genes (Figure 3d). In particular, 86% of suppressed protein-coding genes also had gene promoter histone deacetylation, which is also observed for processed transcripts (69%) and antisense genes (71%). In contrast, 53% of suppressed lincRNA gene promoters are deacetylated, with even lower representation of sense intronic (11%), sense overlapping (38%) and pseudogenes (26%).

Furthermore, activated protein-coding, processed transcripts and antisense genes are strongly associated with increased histone methylation (47%–50%) (Figure 3d). In contrast, we show a moderate relationship between gene suppression and decreased histone methylation for sense intronic (33%), sense overlapping (25%), pseudogenes (17%) and lincRNA (14%), but a weak relationship for protein-coding (2%), antisense (9%) and processed transcripts (11%) (Figure 3d).

Co-occurrence of increased histone acetylation and methylation at activated genes is infrequent and is highest for protein-coding genes (16%) and processed transcripts (16%) compared to all other classes (3%–8%). Decreased histone acetylation and methylation at suppressed genes was almost undetectable (between 0% and 3%). Similar findings were observed for HAECs stimulated with TSA (Figure S2d).

**Figure 4:**
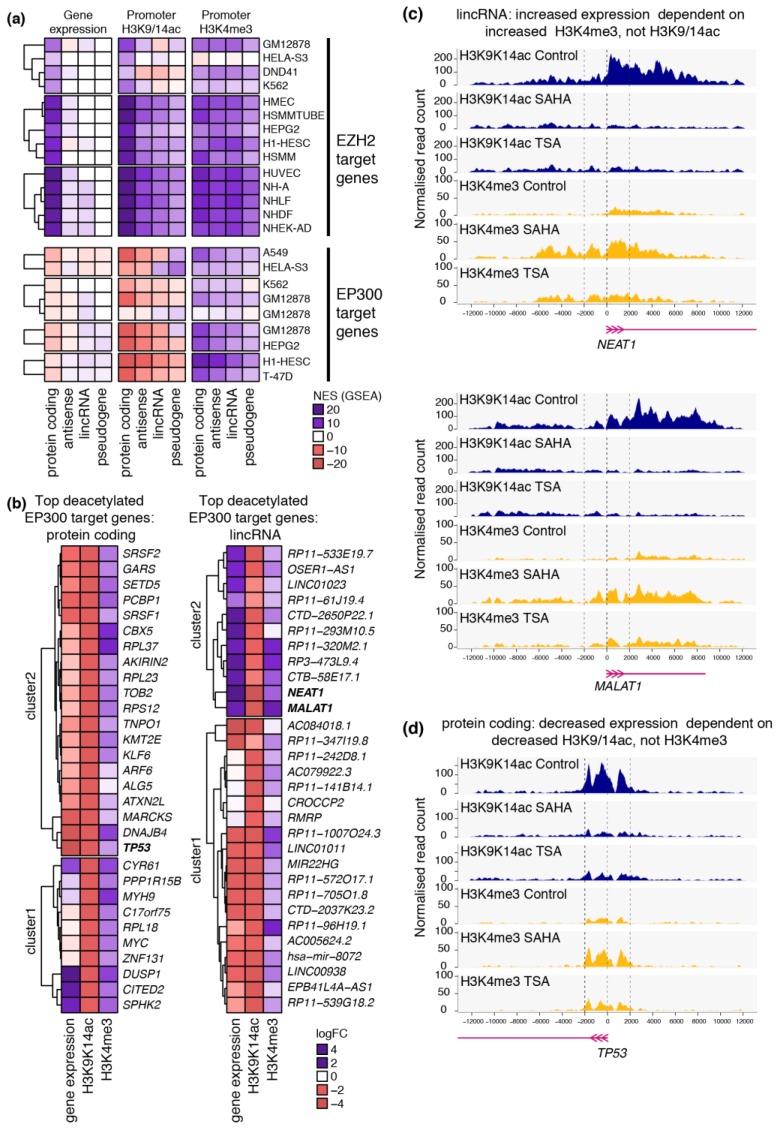
ENCODE-TFBS analysis identified enrichment of EP300 at deacetylated ncRNA promoters. GSEA-ENCODE analysis was used to determine enrichment of transcription factors and chromatin-modifying enzymes at genes regulated by SAHA in HAECs. (**a**) A heatmap showing the normalised enrichment score (NES) for gene sets of EZH2 and EP300 target genes in multiple cell types for coding and non-coding gene expression and histone modifications in HAECs stimulated with SAHA; (**b**) Heatmaps showing logFC of the top EP300 target genes (protein-coding and lincRNA) deacetylated by SAHA and the logFC of the corresponding gene expression and H3K4me3 at gene promoters. H3K9/14ac (blue) and H3K4me3 (yellow) profiles are plotted for the lincRNAs (**c**) *NEAT1* and *MALAT1* and (**d**) *TP53* in control HAECs and those stimulated with SAHA and TSA. Profiles are plotted as the normalised read count and the horizontal axis represents the region surrounding the gene promoter in base pairs.

Next, we combined GSEA with transcription factor binding site gene sets derived from the ENCODE project to study the role of chromatin-modifying enzymes in regulating gene suppression and activation following HDAC inhibition (summarised in Table S1). Protein-coding gene activation and increased histone acetylation and methylation were strongly enriched at genes generally suppressed by Enhancer of zeste homolog 2 (EZH2) (Figure 4a). In contrast, ncRNAs and lincRNAs regulated by EZH2 were associated with increased acetylated and methylation, but not gene activation.

Consistent with our previous reports [9], protein-coding genes regulated by the histone acetyltransferase EP300 were generally suppressed by SAHA (Figure 4a) and TSA (Figure S4) stimulation and subject to promoter histone deacetylation. Strikingly, we show that EP300 target genes were also associated with an increase in H3K4me3 at the gene promoter. In contrast, while lincRNAs regulated by EP300 are also subject to histone deacetylation, these genes are associated with increased gene expression associated with promoter H3K4me3.

We identified complex interactions between histone modifications, chromatin-modifying enzymes and ncRNA gene expression induced by HDAC inhibition (Figure 4b). For example, we observe increased expression of EP300-dependent lincRNAs *MALAT1* (Figure 4c) and *NEAT1* (Figure 4d) despite a strong reduction in histone acetylation both at the gene promoter and across the gene body. The activation of *MALAT1* and *NEAT1* was associated with increased histone methylation rather than a classical acetylation-dependent pathway. In contrast, the protein-coding gene *TP53*, also regulated by EP300, is subject to gene suppression associated with histone deacetylation, despite an increase in histone methylation (Figure 4d).

## 3. Discussion

Pharmacological HDAC inhibitors are a diverse group of drugs that regulate gene expression. SAHA, romidepsin and panbinostat are currently Food and Drug Administration (FDA) approved for the treatment of haematological malignancies [7]. In addition, HDAC inhibitors have potential applications for non-malignant disease: initial pre-clinical studies show efficacy of multiple HDAC inhibitors for the treatment of cardiovascular disease and diabetes [12,13,14].

The advent of multiple consortiums extensively profiling histone modifications and transcription factors across diverse human cells and tissues have advanced the study of chromatin modifications and their impact on gene expression. Recently, the complex epigenetic mechanisms involved in the regulation of lincRNA were profiled across diverse human tissue types as part of the National Institutes of Health (NIH) Roadmap Epigenomics Project [6]. While there have been reports that HDAC inhibitors can modulate the expression of individual ncRNAs, the genome-wide expression patterns and epigenetic mechanisms involved remain poorly understood. In this study, we show that HDAC inhibitors SAHA and TSA are potent regulators of ncRNA expression, which is driven by complex epigenetic mechanisms that are dependent on the ncRNA class.

In particular, HDAC inhibitor mediated epigenomic regulation of antisense and processed transcript ncRNA groups is similar to the regulation of protein-coding genes, but differs from lincRNA, sense overlapping genes, sense intronic genes and pseudogenes. Histone deacetylation is a stronger driver of gene suppression for antisense, processed transcript and protein-coding genes compared to other ncRNA classed.

Surprisingly, we show that bivalency following HDAC inhibition occurs infrequently. Instead, we often find opposing histone modifications occurring together. For instance, we show that the expression of the lincRNA *MALAT1*, which is regulated by EP300, is increased despite a strong reduction in histone acetylation across the gene promoter and gene body. This is associated with an increase in promoter histone methylation instead. *MALAT1* is a key regulator of endothelial cell function and vascular growth [11], and though its expression is activated by HDAC inhibition in primary endothelial cells, the mechanisms involved are more complex than the classical paradigm of histone hyperacetylation. Furthermore, while this study has focused on endothelial cell biology, modulation of ncRNA expression by HDAC inhibition has been observed in multiple cell types [15,16,17]. In particular, TSA modulates the expression of ncRNAs in hypertrophic cardiac tissue [18], and has also been shown to regulate histone acetylation at cardiac gene promoters [13]. However, the direct modulation of ncRNA expression by altered histone acetylation in TSA-mediated reversal of cardiac hypertrophy has yet to be investigated.

Consistent with recent studies, we identify that the effects of SAHA and TSA on gene expression and histone modifications are very similar [9]. This is possibly because these drugs are both hydroxamic acids and therefore share similar structures. It is not yet clear whether other HDAC inhibitor drug classes would exert a similar effect. Furthermore, while public databases such as the ENCODE project can be useful to determine transcription factors associated with gene expression changes, there is limited data available about the genome-wide binding profiles of HDAC enzymes. In particular, public consortiums may be informative of HDAC inhibitor-mediated histone independent mechanisms of gene expression and suppression, especially the altered activity of transcription factors.

While we explored changes to H3K9/14ac and H3K4me3, other histone modifications, such as H3K4me1 and H3K27ac, have been implicated in the regulation of lincRNA expression, specifically at gene enhancers [6]. In particular, H3K4me1 was identified as a key histone modification involved in the regulation of tissue-specific lincRNA expression [6]. Furthermore, H3K27me3 is often associated with the suppression of lincRNA during cell differentiation [6]. Further studies exploring changes to these histone modifications, coupled with RNA sequencing, will be highly informative.

In summary, we have identified complex histone modifications involved in the regulation of ncRNA expression mediated by HDAC inhibition. Given the importance of ncRNAs in the regulation mechanisms relevant to human disease and the increasing use of HDAC inhibitors in the clinic, a better understanding of the regulatory mechanism may enhance rational drug design and development of new therapeutic targets and applications.

## 4. Materials and Methods

Data access and preliminary analysis: RNA-seq and ChIP-seq datasets were accessed from Gene Expression Omnibus (accession number: GSE37378). In this study, primary human aortic endothelial cells were stimulated with 500 nM TSA or 2 μM SAHA for 12 h. Reads with quality scores of less than 30 were trimmed from the fastq file. Fastq files were aligned to the hg19 genome from Ensembl (GRCh37 release 75) using STAR for RNA-seq and Burrows-Wheeler Aligner (BWA) [19] for ChIP-seq. For RNA-seq, a matrix was generated to summarise total gene reads and edgeR software was used to determine differential gene expression [20]. For ChIP-seq, a matrix was generated based on reads located within gene promoters (defined as 2 kb either side of the transcription start site, TSS) and changes to histone modifications were determined using edgeR [20]. Significance is defined as FDR *p* value < 0.05.

Gene classes: gene classes (protein-coding and the various non-coding classes) were defined using the Ensembl gene biotype classification [2].

Statistics: Pearson’s correlation coefficient (r) was calculated in R using the ‘cor’ function. Fisher’s exact test was used to determine the relationship between histone modifications and gene expression for different classes. The four quadrants were defined as: (1) genes in defined class with defined histone modification; (2) genes in defined class without defined histone modification; (3) genes not in class with defined histone modification; and (4) genes not in class without defined histone modification. Results are reported as the log2 of the odds ratio with 95% confidence intervals.

Transcription Factor Analysis Gene Set Enrichment Analysis (GSEA) was used to determine enrichment of transcription factor and chromatin binding protein at genes regulated by HDAC inhibition [21]. For each gene transcript, expression, histone acetylation and histone methylation were assigned a score based on the negative log10 of the *p* value multiplied by the sign of the fold change (–logP x signFC). GSEA was run using classical scoring with 1000 permutations. Only gene sets between 30 and 7000 genes were included in the analysis. Gene sets of transcription factor target genes were generated from ENCODE transcription factor binding site (TFBS) ChIP-seq bed files [22]. TFBS bed file represents a list of genome regions targeted by transcription factor binding. Target genes were defined as possessing a TFBS within 3 kb either side of the TSS.

Plot generation: MA plots were generated in R by plotting the fold change against the logCPM. The logCPM represents the relative expression of the gene (or, for ChIP-seq, relative level of histone modification in the defined region) across all samples and is the logged counts-per-million. Correlation plots were generated in R by plotting the logFC of two different experiments. Pearson’s correlation coefficient and a linear model are also included. Heatmaps were produced in R using the bioconductor package ComplexHeatmap. Histone modification profiles were plotted in R from read counts extracted from bedGraph files.

## Supplementary Materials

The following are available online at www.mdpi.com/2311-553X/2/2/4/s1, Figure S1. Stimulation by SAHA and TSA lead to robust ncRNA expression, Figure S2. Epigenetic regulation by TSA is ncRNA class-specific, Figure S3. Epigenetic regulation of ncRNA by HDAC inhibition is class-specific, Figure S4. ENCODE-TFBS analysis identified enrichment of EP300 at deacetylated ncRNA promoters, Table S1: Summary of results for ENCODE-TFBS results for EP300 and EZH2 dataset in HAECs stimulated with SAHA and TSA.

## Abbreviations

The following abbreviations are used in this manuscript:

ChIP-seq	Chromatin immunoprecipitation sequencing
ENCODE	Encyclopedia of DNA Elements
FDA	Food and Drug Administration
FDR	False Discovery Rate
GSEA	Gene Set Enrichment Analysis
H3K27ac	Histone 3 lysine 27 acetylation
H3K27me3	Histone 3 lysine 27 trimethylation
H3K4me1	Histone 3 lysine 4 monomethylation
H3K4me3	Histone 3 lysine 4 trimethylation
H3K9/14ac	Histone 3 lysine 9 and 14 acetylation
H3K9ac	Histone 3 lysine 9 acetylation
HAECs	Human Aortic Endothelial Cells
HAT	Histone acetyltransferase
HDAC	Histone deacetylase
lincRNA	long intergenic non-coding RNA
logCPM	Log of the counts per million
logFC	log of the fold change
miRNA	microRNA
ncRNA	non-coding RNA
NIH	National Institute of Health
ORF	Open Reading Frame
RNA-seq	RNA sequencing
SAHA	Suberanilohydroxamic acid
TFBS	Transcription Factor Binding Sites
TSA	Trichostatin A
