# Supplementary material for: HDAC Inhibition in Vascular Endothelial Cells Regulates the Expression of ncRNAs
Source: Noncoding RNA. 2016 May 25;2(2):4. doi: 10.3390/ncrna2020004 (PMC5831901; doi:10.3390/ncrna2020004)
Supplement: Supplementary file 1 [file ncrna-02-00004-s001.zip › ncrna-133008-supplementary-final/ncrna-133008-supplementary-figures.docx]

Supplementary Materials: HDAC Inhibition in Vascular Endothelial Cells Regulates the Expression of ncRNAs

Haloom Rafehi and Assam El-Osta

#
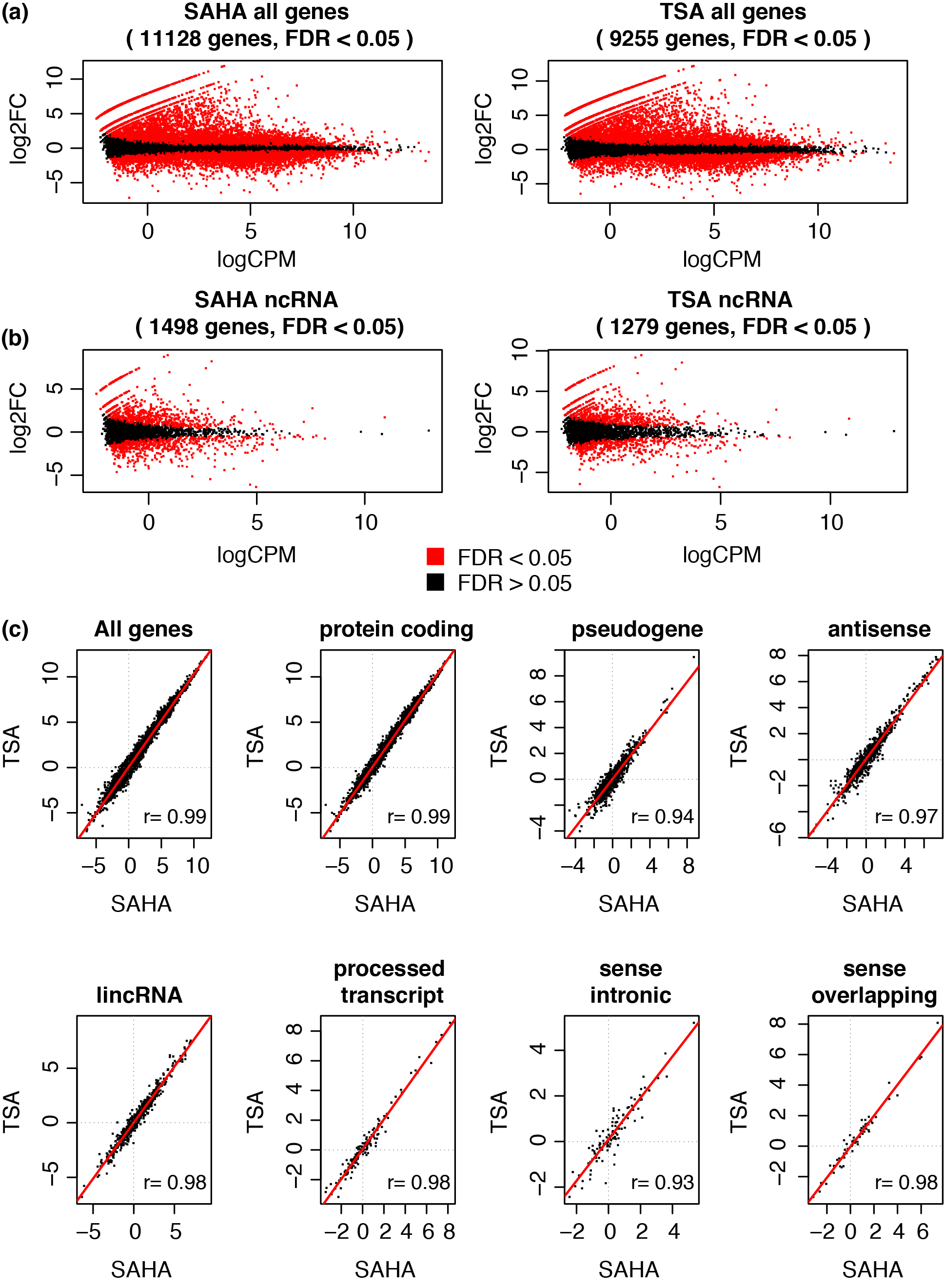


**Figure S1.** Stimulation by SAHA and TSA lead to robust ncRNA expression. MA plots show the relationship between changes (logFC, log2 of the fold change) and the relative read count (logCPM) for (**a**) all genes and (**b**) ncRNA genes in response to SAHA and TSA. Red points indicates FDR *p* value < 0.05, and black indicates FDR *p* value > 0.05. The number of genes FDR < 0.05 is shown in parenthesis on top of each plot; (**c**) scatterplots plots of the fold changes (logFC) show the correlation of gene expression for different gene classes in response to TSA and SAHA. Linear model is shown in red and the Pearson’s correlation value is reported for each plot.

#
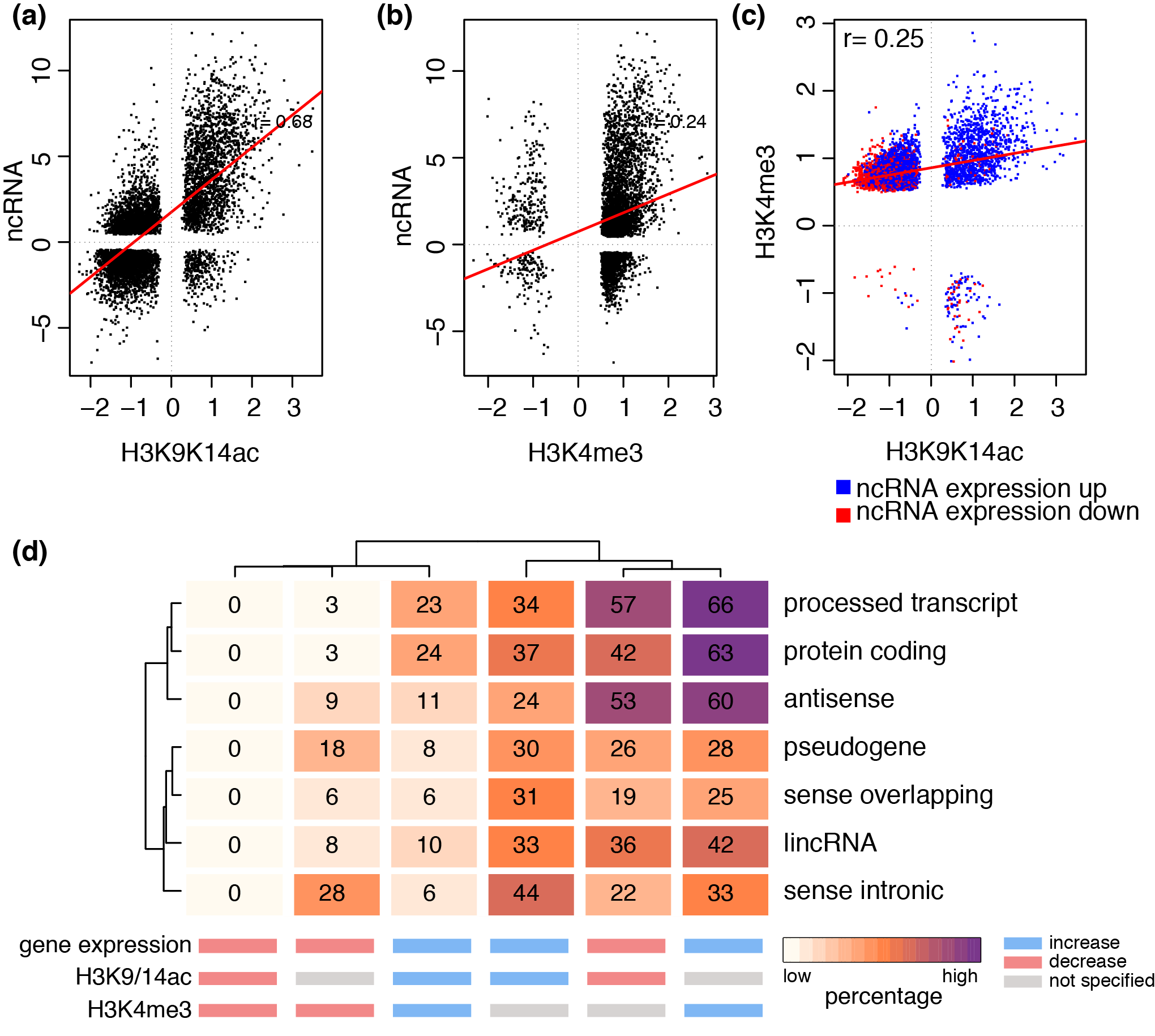


**Figure S2.** Epigenetic regulation by TSA is ncRNA class-specific. Scatterplots plots of the fold changes (logFC) show the correlation between ncRNA expression (FDR *p* value < 0.05) and corresponding promoter (**a**) H3K9/14ac and (**b**) H3K4me3 (FDR *p* value < 0.05) following stimulation with TSA; (**c**) a scatterplot of the fold changes (logFC) shows the correlation between differential promoter H3K9/14ac and H3K4me3 (FDR *p* value < 0.05) for ncRNA genes that are either activated (blue) or suppressed (red) (FDR *p* value < 0.05). Linear model is shown in red and the Pearson’s correlation value is reported for each plot; (**d**) a heatmap showing the percentage of activated or suppressed genes that overlap with increased or decreased histone modification (defined on horizontal axis) for protein coding genes and six ncRNA classes (vertical axis).

#
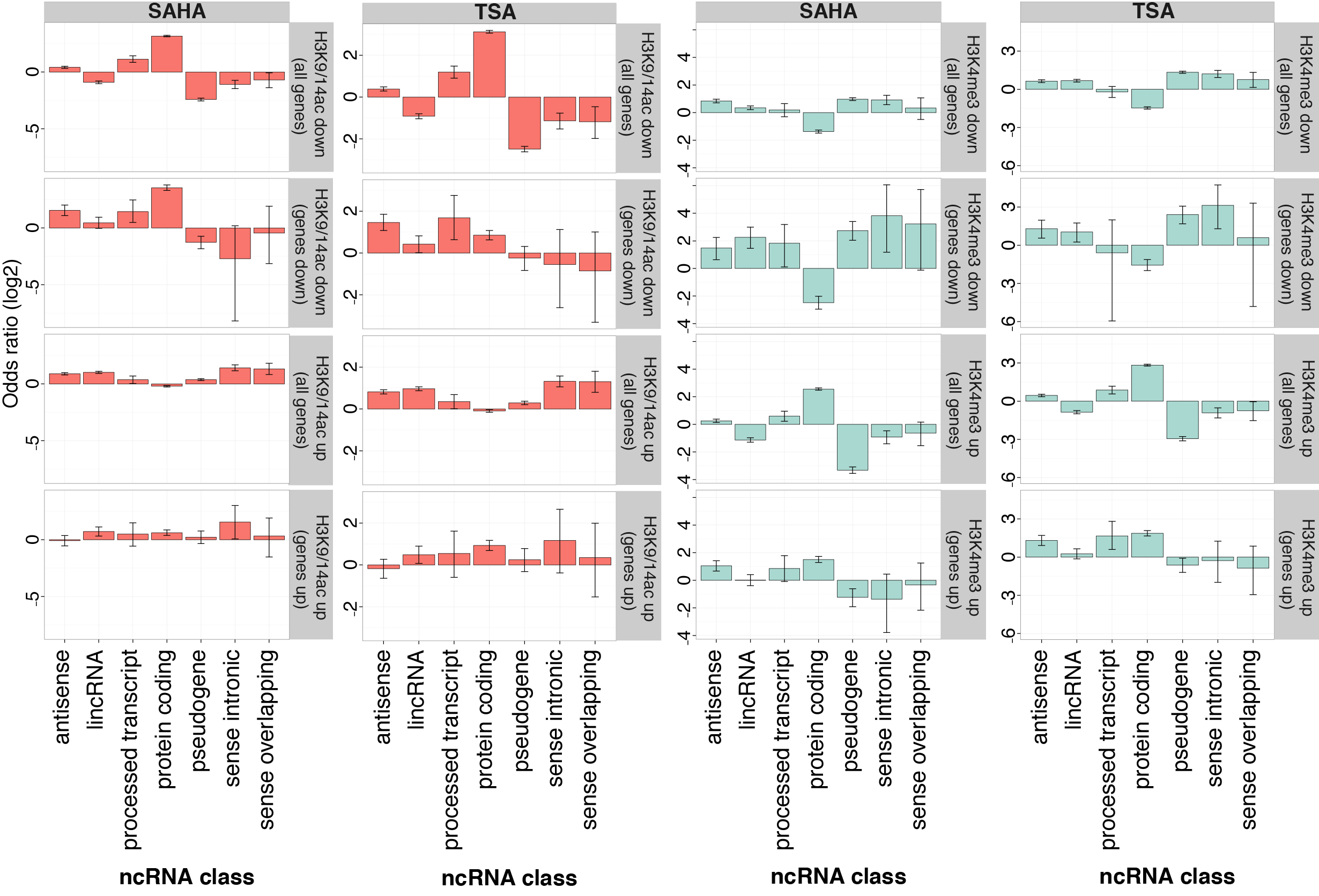


**Figure S3.** Epigenetic regulation of ncRNA by HDAC inhibition is class-specific. Fisher’s exact test was used to determine the relationship between histone modifications and gene expression for different gene classes following stimulation with SAHA or TSA. Results are reported as the log2 of the odds ratio with 95% confidence intervals.

#
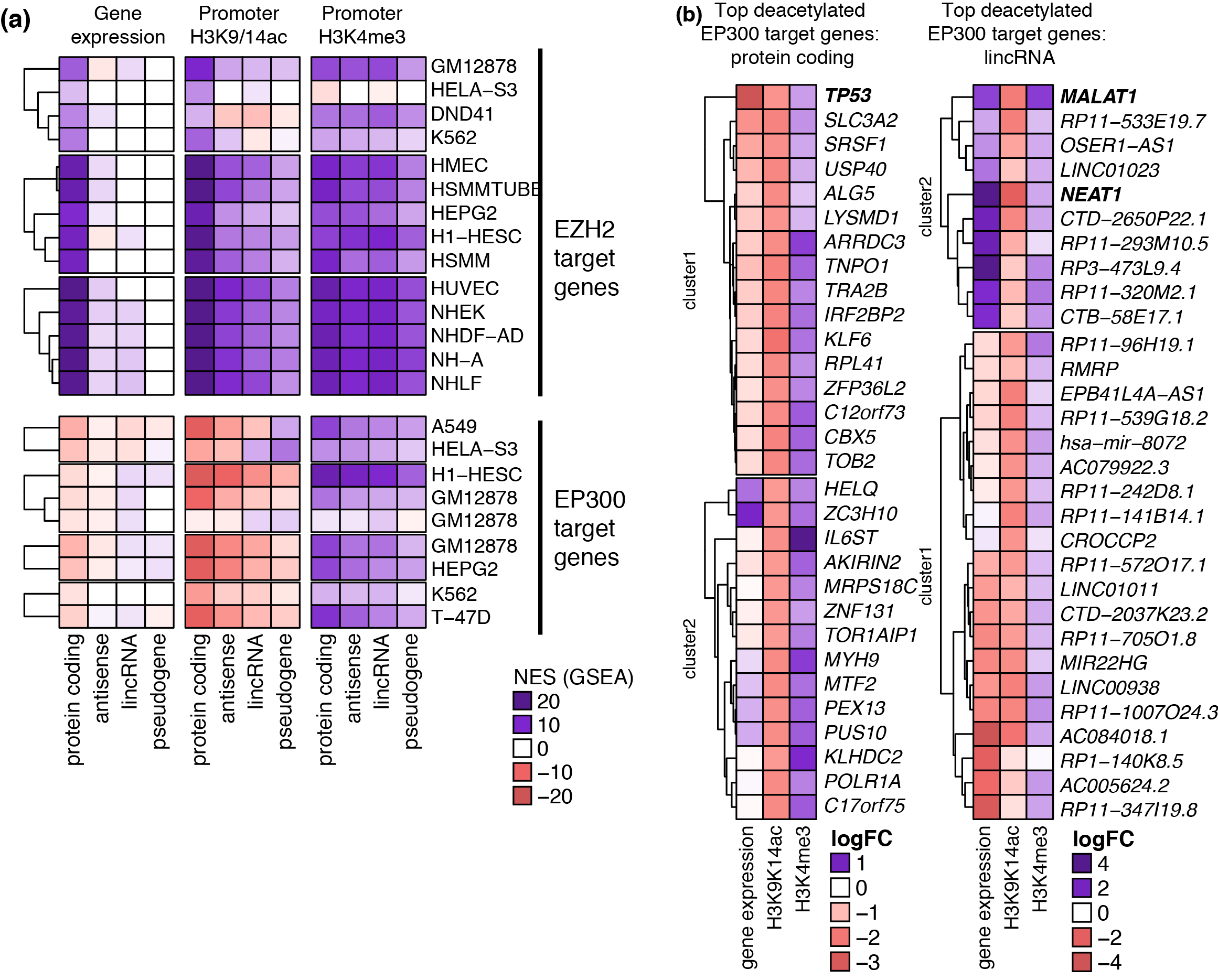


**Figure S4.** ENCODE-TFBS analysis identified enrichment of EP300 at deacetylated ncRNA promoters. GSEA-ENCODE analysis was used to determine enrichment of transcription factors and chromatin-modifying enzymes at genes regulated by TSA in HAECs. (**a**) A heatmap showing the normalised enrichment score (NES) for gene sets of EZH2 and EP300 target genes in multiple cell types for coding and non-coding gene expression and histone modifications in HAECs stimulated with TSA; (**b**) heatmaps showing logFC of the top EP300 target genes (protein coding and lincRNA) deacetylated by TSA and the logFC of the corresponding gene expression and H3K4me3 at gene promoters.
